# An Effective Spectrum Handoff Based on Reinforcement Learning for Target Channel Selection in the Industrial Internet of Things

**DOI:** 10.3390/s19061395

**Published:** 2019-03-21

**Authors:** Stephen S. Oyewobi, Gerhard P. Hancke, Adnan M. Abu-Mahfouz, Adeiza J. Onumanyi

**Affiliations:** 1 Department of Electrical Electronic and Computer Engineering, University of Pretoria, Pretoria 0083, South Africa; ghancke@gmail.com (G.P.H.); AAbuMahfouz@csir.co.za (A.M.A.-M.); adeiza1@yahoo.com (A.J.O.); 2 Computer Science Department, City University of Hong Kong, Hong Kong, China; 3 Modelling and Digital Science, Council for Scientific and Industrial Research, Pretoria 0083, South Africa

**Keywords:** spectrum handoff, industrial-internet of things, cognitive radio, dynamic spectrum access, reinforcement learning, channel selection strategy

## Abstract

The overcrowding of the wireless space has triggered a strict competition for scare network resources. Therefore, there is a need for a dynamic spectrum access (DSA) technique that will ensure fair allocation of the available network resources for diverse network elements competing for the network resources. Spectrum handoff (SH) is a DSA technique through which cognitive radio (CR) promises to provide effective channel utilization, fair resource allocation, as well as reliable and uninterrupted real-time connection. However, SH may consume extra network resources, increase latency, and degrade network performance if the spectrum sensing technique used is ineffective and the channel selection strategy (CSS) is poorly implemented. Therefore, it is necessary to develop an SH policy that holistically considers the implementation of effective CSS, and spectrum sensing technique, as well as minimizes communication delays. In this work, two reinforcement learning (RL) algorithms are integrated into the CSS to perform channel selection. The first algorithm is used to evaluate the channel future occupancy, whereas the second algorithm is used to determine the channel quality in order to sort and rank the channels in candidate channel list (CCL). A method of masking linearly dependent and useless state elements is implemented to improve the convergence of the learning. Our approach showed a significant reduction in terms of latency and a remarkable improvement in throughput performance in comparison to conventional approaches.

## 1. Introduction

In recent times, there has been a growing interest in the emergence of IoT as an enabling technology in the realization of an energy efficient communication in the Industry 4.0 paradigm [1,2]. As a result, industrial-IoT (IIoT) has become one of the most researched topics in the last decade [3,4]. However, the stringent QoS requirements of the industrial application scenarios, e.g., reliability, timeliness, and robustness, coupled with some IoT challenges have slowed down the realization of IIoT [5,6,7]. Table 1 shows the real-time and QoS requirements of industrial wireless communications, and applications [5,8]. Moreover, there are some challenges associated with IoT which are inimical to the realization of IIoT like [9,10,11]: (1) spectrum, memory, energy and computational limitations; (2) security challenges and security requirements e.g., cyber-security and privacy, authorization, authentication, integrity, and confidentiality [12,13]; (3) fault tolerance [14], and (4) scalability with legacy devices and (5) interoperability issues.

A recent approach of mitigating these challenges, particularly the problem of spectrum limitations (licensed and unlicensed bands) in industrial application scenarios, has been to incorporate cognitive radio (CR) technology into IIoT. CR is a new paradigm which mitigates spectrum scarcity and its associated challenges by allowing CR users to temporarily make use of the licensed spectrum of the primary user (PU) if the licensed spectrum is unoccupied by a PU. CR can be used to improve spectrum utilization [1] in IIoT due to the massive potential benefits of CR, and the enormous challenges of IIoT [15]. The objective of CR-IIoT is to mitigate the aforementioned challenges of IoT which limit the real-time capabilities of IIoT technology. However, due to the high priority of a PU a low priority CR user must vacate a channel previously occupied by the CR user when a PU arrives on the channel. This process is known as spectrum handoff (SH). In addition, the CR user must initiate an interrelated but entirely different process known as channel selection strategy (CSS) to determine and to select a new suitable channel (target channel) to continue its ongoing transmission. However, finding a new channel to continue unfinished transmission is a non-trivial task. 

From a technical point of view, target channels can be preselected before an SH is triggered (proactive SH) or can be selected by instantaneous wideband scanning of the licensed channel at the instant an SH is triggered (reactive SH). However, in the proactive SH, preselected channels may be outdated at the instant an SH is initiated, whereas, in the reactive SH, the interval spent in sensing the licensed spectrum for idle channels incurs an additional time, resulting in increased SH delays as well as a high communication latency [16]. To mitigate these challenges, the IEEE 802.22 standard specifies that a CR user should keep a candidate channel list (CCL) [17]. However, the IEEE 802.22 standard did not define a specific algorithm for selecting a target channel from the backup channel list (BCL), or for building a BCL from the CCL [18]. Similarly, to encourage novelty, the sorting/the ordering of the channels in the CCL is left open by the IEEE 802.22 standard. This is significant because when a target channel is selected arbitrarily based on channel availability alone, there is a likelihood of selecting a channel with poor channel quality [18]. Moreover, when poor channels are selected it leads to degraded network performance. Also, high communication latency is introduced because the node keeps repeating the process of channel selection in an attempt to select a channel with better quality. 

To mitigate such possibilities and to improve the effectiveness of an SH, in this paper, we propose a CSS based on a reinforcement learning (RL) algorithm [19]. Q-learning is the RL method chosen in this work. This is because Q-learning has been shown to be an effective RL algorithm in scenarios where several parameters and policies are adjusted simultaneously such as the IEE 802.22 [20]. Q-learning also allows CRs to adapt to their environment efficiently without complete knowledge of the interaction between the adjusted parameters and policies [20]. The proposed technique integrates two Q-learning algorithms into the channel selection process to improve the target channel selection. As a result, the proposed technique does not only consider the absence of PU activity on the channel, but it also considers the quality of the channel to analyse, to generate, and to sort the channels in the CCL.

Similarly, to implement an SH scheme with a minimum channel-switching delay in the event of an interruption by a PU to an unfinished CR user transmission, it is important to quickly decide in which order to do a wideband sensing to select a suitable channel from a potential list. In this work, to improve the convergence time of the RL algorithm in selecting channel with good quality, only space elements that are relevant to task learning are retained from sensing information. This work presents a novel SH scheme with a CCL based on an RL algorithm known as SH+RL scheme that holistically takes into consideration the CSS, spectrum sensing, as well as communication delays. The main contributions of this paper are summarized as follows:

(1) We propose an SH scheme that separates spectrum sensing, profiling, sorting, and ranking of target channels from channel-switching time of CR user, this approach minimizes the channel-switching delay.
(2) We propose a network model that achieves low latency by introducing a gateway that only performs the spectrum sensing function (SSF) to eliminate the time spent in spectrum sensing from channel-switching time.
(3) We introduce RL into the design of the proposed CSS to improve the quality of channels selected for an SH to reduce avoidable SHs and to improve throughput performance of the network.
(4) We use a masking method to mitigate the curse of dimensionality to reduce the convergence of the Q-learning algorithms. Then we develop a BCL to prevent collision between multiple CR users attempting to use the same channel, this improves the throughput performance of our approach.

The remainder of this paper is organized as follows: Section 2 reviews related works. In Section 3, we introduce our simulation scenario and considerations. Then, Section 4 gives a detail description of the proposed SH+RL scheme. While Section 5, describes the simulation setup. In Section 6, results of the simulation are presented and discussed and finally, Section 7 concludes this paper.

## 2. Related Works

IIoT applies IoT to industry by presenting great opportunities for new applications having the capacity to improve productivity in industry [21]. An important feature of IoT technology which drives IIoT is the collaboration among heterogeneous IoT devices. However, the dense deployment of sensor nodes for IoT often leads to severe transmission and resource allocation challenges. Therefore, interoperability between diverse communication devices and efficient communication between sensing devices are significant challenges faced by IIoT [22]. Several attempts at solving interoperability problems in industry have been proposed in the literature. For example, time-synchronized standards, like the IEEE802.15.4-2006 LoWPAN, which combines random access and dedicated timeslots, for real time transmission have been developed. Other protocols like the 6LoWPAN and constrained application protocols (CoAP) have advanced the development of IoT devices that are both interoperable and standard-compliant [23]. On the other hand, in [24] the authors proposed a multi-network framework for improved communication efficiency in IIoT. A middleware support is proposed in [25] by deploying fog computing to provide local processing support to reduce latency for robots in the industry.

Another critical challenge of IIoT is reliability of the data sensed and transmitted by the industrial wireless sensor network (IWSN). IoT devices depend on sensed data to take action, therefore, packet delivery reliability is a critical challenge in IIoT. In [26], the authors proposed a loss-tolerant communication protocol that aids message exchange with high resilience and low packet loss. The security of the data exchange and authentication between IoT devices is the approach adopted in [27] to ensure high packet delivery reliability. In order to ensure an efficient real time and reliable communication for construction vehicles, with a balance between cost and monitoring scale, in [28], an industrial-QoS-oriented transparent transmission protocol was developed. However, due to the dense deployment of sensor nodes in IoT, the problem of packet delivery reliability is further aggravated by the scarcity of radio frequencies for transmitting the massive volume of exchanged data. One approach to guaranteeing high efficiency spectrum utilization in high volume data transmission is by integrating CR into IoT. With CR, different channels can be used to transmit packets simultaneously, and by handoff, the occupied channels can be vacated when a PU arrives requesting access [29]. For example, sensor nodes can send data to the IoT devices or to a data centre using DSA with various frequencies. Remarkably, previous works on spectrum sensing for channel classification in IIoT are scarce in literature. In [30], a handoff management scheme with a QoE-driven channel allocation strategy, in cognitive 5G cellular network for seamless multimedia was developed. In this work, channel usage statistical information was used to evaluate channel quality, to maintain a ranking index and to minimize SH. A priority-based channel allocation strategy to share channels to users based on their QoE requirements was developed to enhance channel utilization. The results highlighted an improvement with respect to channel utilization and received video quality. However, in cognitive radio network (CRN), channel availability is the sole metric used in channel selection [31]. Similar to CRNs, and specifically in CR-IIoT, little or no attention has been given to channel quality as a key metric in channel selection for SH strategies. Especially, in CR-IIoT, where accurate statistical information of the PU, and the channel conditions are required in characterizing the spectrum and in selecting the best channels for efficient high volume data transmission. However, some literatures have considered channel quality as well as channel availability as important metrics for channel selection in CRNs. In [32], authors showed that channel availability as well as channel quality are the two significant metrics that should be considered when designing an efficient CSS. In their work, an L-CAQ channel selection scheme was design, which selects channel that jointly maximizes channel availability probability as well as channel quality. Similarly, in [33] an adaptive channel selection method was developed for hierarchical cluster-based CRNs. In their work, when a legacy system begins to experience decreasing channel quality, channels with the highest ranking position based on a predefined sensing policy are selected as possible backup channels. In [18], by taking into account behavior of PU on the channel and the received signal strength indicator (RSSI) value of the channel, a channel selection method based on fuzzy logic was developed for IEEE 802.22. The results presented in [18] are helpful in selecting channels as target channel from a backup channel list. The work in [34], proposed a link-aware channel selection technique, which considers the impact of unreliable wireless link. As a result, a scheme which reroutes transmission in lossy links was developed for body networks in their work. 

To the best of our knowledge, this work constitutes the first attempt, where CR has been applied in solving the problem of high volume data transmission in real time in a IIoT scenario. Conversely, there have been works in the literature on the use of the RL algorithm and spectrum sensing for finding and sorting vacant channels for CRNs. In [35], a channel classification system using Q-learning+ algorithm is proposed where a RL algorithm based on the historical behavior of PU and channel conditions is deployed to create channels in the CLL to enhance dynamic spectrum allocation. Q-learning uses a reward system to define the order in which channels are analysed. The reward for each channel is calculated after every transmission on the channel. The major drawback of this approach is that the channel chosen at the beginning of a transmission may not have been the best channel since learning can only be implemented on channels in which CR users have previously transmitted. However, in our work, only space elements that are relevant to task learning are retained from sensing information to improve the convergence of RL learning. This is to reduce the delays in selecting the best channel during transmission interruptions by the arrival of the PU, so that data can be exchange in real time.

## 3. System Model

### 3.1. Assumptions

For the remainder of this paper, we consider a multiple-node IIoT model consisting of both PUs and CR users existing side-by-side, with partially overlapping coverage areas. The CR users are essentially IIoT nodes with CR capabilities. We assume that PUs communicate with each other using N channels licensed bands, through a synchronous slot structure. We consider that each channel has a bandwidth $B_{i}$ where i = 1, 2, 3,…, N and that the network state is designated by time slot *t*, such that [35]:

(1) $$t=\left[S_{1}\left(t\right),\ldots \ldots S_{N}\left(t\right)\right]$$

where $S_{1\;}\left(t\right)$ ∈ {0 (unoccupied), 1 (occupied)}. We assume that default CR communication starts in the industrial, scientific, and medical (ISM) band, henceforth known as the unlicensed band using single hop connection. However, we consider that CR users can communicate with other CR users through overlay transmission by opportunistic transmission in the licensed band. We consider that channel capacity is characterized by the signal-to-interference-plus-noise ratio received at the receiver of CR users [36] as shown (2):

(2) $$sinr_{i}=\frac{h_{i,j}\left(t\right)x_{j}\left(t\right)}{\sum_{j}h_{i,j}\left(t\right)x_{j}+n_{i}\left(t\right)}$$

where $x_{j}\left(t\right)$ represents the signal transmitted from CR user j, $h_{i,j}\left(t\right)$ is the gain of the channel for the transmission from CR user j to i and $n_{i}\left(t\right)$ stands for the noise at CR user i. However, since white spaces do exist up to 60% of the time in the ISM band due to the bursty nature of the traffic in IIoT, which can be exploited by CR users for their communication [5,37]. In this work, different from existing CR-related models, we assume that white space opportunities exist in both licensed and unlicensed bands for industrial wireless communication. 

### 3.2. Network Model

**Figure 1:**
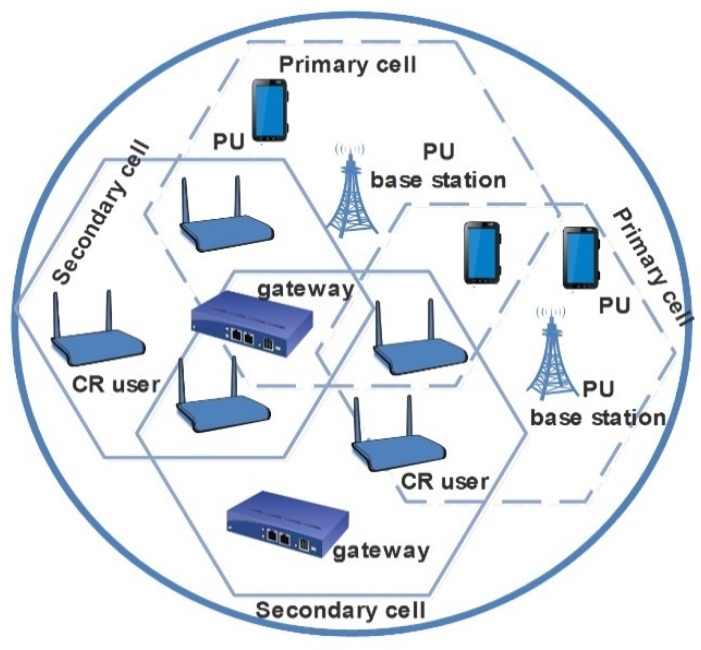
Proposed network model.

The network model in this work is illustrated in Figure 1. It is a cluster-based CR-IIoT network which is based on the IEEE 802.15.4 standard. The network comprises several clusters with a gateway as the cluster-head. In each cluster, a gateway independently performs spectrum sensing and sends its decision to the IIoT devices in its cluster through a common control channel. However, the gateways share information with each other as an ad-hoc cooperative CRN. The following nodes exist in the network: (1) IIoT nodes with CR capabilities (i.e., CR users), (2) PU, and (3) Gateway which are CR-enabled cluster-head (acting as network coordinator in each clusters in the network).

A PU is considered a hidden node if an IIoT node is within the coverage area of the PU. However, if the IIoT node is outside the coverage area of the PU it is a co-existing node. An IIoT node can transmit on the same channel with a co-existing PU node without causing harmful interference to the PU activity. To prevent the problem of hidden node terminal, the gateway keeps a hole information array ϕ of m channels within any radius $r_{j,m}$ of a PU j as shown in (3);

(3) $$\phi =\left\{\psi_{m,n}|\psi_{m,n}\left\{1\left(co-existence\right),0\left(hidden\right)\right\}\right\}$$

where ϕ is an M by N matrix, and $\psi_{m,n}$ is the information that determine if a PU is either a hidden or co-existence node as illustrated in (4);

(4) $$\psi_{m,n}=\{\begin{array}{cc} 1 & r_{n,m}\leq d_{n,j}-r_{j,m}\\ 0 & otherwise \end{array}$$

where $d_{n,j}$ is the distance between an IIoT node n and a PU j, $r_{n,m}$ and $r_{j,m}$ are coverage area of the IIoT node n and PU j respectively. Therefore, the IIoT node must perform an SH immediately as soon as it discovers that a PU occupying a channel is a hidden node to forestall any harm to the PU transmission. 

### 3.3. Channel Modelling

The channel in the industrial environment is non-stationary and can change abruptly due to the characteristics of the IIoT environment. As a result, the received signal power in the industrial wireless environment is not only dependent on the transmitted signal power, the antennas gain, and the distance between the transmitter and the receiver, but also depends on the effects triggered by the environment. In this paper, the industrial wireless environment is modelled as a log-distance path-loss model to predict the path-loss and shadowing effects which are symbolic of the IIoT environment as represented in (5) [37,38]:

(5) $$PL_{i,j}=P_{tx,i}-P_{rx,j\;}=PL_{o}+10\gamma \log_{10}\left(d_{i,j}\right)+X_{g}+\frac{d_{i,j}}{d_{\phi }}\;X_{k}$$

where $PL_{i,j}$ is the path-loss experienced when CR user i is transmitting to CR user j, and $P_{tx,i}$ is the transmitted power by CR user i in dBm, $P_{rx,j\;}$ is the received power by CR user j in dBm. $\;PL_{o}$ is the path-loss in dB at a distance of 1 m, γ is the path-loss exponent (PLE) which specifies the rate at which the path-loss increases with distance (PLE value for obstructed industrial environment is 2 to 3, $d_{i,j}$ is the distance between the transmitter and the receiver in (m), $X_{g}$ is a zero-mean Gaussian distributed random variable reflecting signal attenuation in dB, with a standard deviation of −σ, $X_{k}$ is the obstacle loss in dB, $d_{\phi \;}$ is included to specify the distance between two obstacles in (m).

## 4. Design of the Proposed Scheme

In this section, a detailed description of the SH+RL scheme is provided. The SH+RL scheme consists of a number of separate but interconnected tasks which are performed by the different nodes in the network. These tasks include: (1) selecting channels for the CCL, which is performed by the gateway, (2) creating an exclusive BCL for each CR user in the network, which is also performed by the gateway, (3) conditions for SH, and (4) decision-making respectively which are tasks implemented on/by the individual CR users in the network.

### 4.1. Selecting Channels for the CCL

In the network, the IIoT devices deploy DSA to transmit opportunistically in the PU licensed band. Then, the IIoT devices occasionally receive channel updates from the gateways which act as network coordinators in a star topology [39]. The gateway performs only the SSF, and broadcasts channel updates periodically. The IIoT device with the highest residual time (waiting time), then selects the first channel in the CCL to transmits its packets. This is to mitigate the drawback of overlapping the sensing and analysis of the spectrum with the actual IIoT device’s transmission. By deploying two Q-learning algorithms the gateway performs the following functions before an SH is triggered to reduce communication latency; (1) performs spectrum sensing to create the CCL, (2) sorts and ranks the available channels in the CCL (3) keeps statistical history of PU’s communication pattern, and (4) updates a BCL for each CR user in the network. 

Q-learning is an RL technique that allows a proxy to learn the optimal policy to follow in a known environment [17,38,40,41]. A set of possible states $X=x_{1},x_{2},\;x_{3},\ldots ,\;x_{n}$, and a set of actions $A=a_{1\;},a_{2\;},a_{3},\ldots ,a_{n,}$ describing the environment is defined in this model. A proxy selects an action $a_{t}$ to perform in the current state $x_{t}$ such that $a_{t}\in A$ and $x_{t}\in X$ respectively. Based on this action, a transition to a new state $x_{t+1}=y$ according to the probability $P_{xy}\left(a\right)$ where  y∈X is triggered. This new state permits the proxy to compute a reward. In the end, the aggregate reward computed by the Q-learning algorithm, is used to make a decision. The expected aggregate reward $V^{\pi }\left(x\right)$ due to a set of actions and rules which causes transitions in a set of states is calculated in (6) [38]:

(6) $$V^{\pi }\left(x\right)=\underset{N\to \infty }{\lim}E\;\left(\overset{N}{\underset{t=1}{\sum }}r_{t}^{\pi }\left(x\right)\right)$$

where $r_{t}^{\pi }\left(x\right)$ is reward calculated at time t after starting from state x  and by following policy π. 

Since our objective is to select channels with a high probability of being vacant and as well as having good channel quality, the objective function can be represented by a set of actions and rewards. Hence, we propose the use of the Q-learning algorithm to implement the CSS problem.

### 4.2. Integrating Two Reinforcement Learning Algorithms to Perform Channel Selection

We assume that each gateway in a cluster is a proxy implementing the RL algorithms through an action-selection strategy such that actions a∈A corresponds to all possible channel selection classifications. The algorithms have a sole objective of maximizing the likelihood of selecting the channels with the highest probability of being vacant as well as having good quality as the channels with the best characteristics. The gateway implements two Q-learning algorithms during the spectrum sensing period. 

The spectrum sensing period is composed of two parts; in the first spectrum sensing part, the gateway analyses the utilization profile of the channels through the first algorithm called the Historical occupancy learning to determine which channels are vacant or occupied. Then, in the second part, for channels that have been determined vacant in the first part, the gateway uses the mean RSSI value to obtain information about the conditions of the channels through the second algorithm known as the Channel Conditions Learning [17]. 

In the sensing periods (t), all the potential channels are scanned by the gateway. The total duration of the SSF is split into T spectrum sensing periods with interval $t_{D}$, such that $t_{D}=1,\;2,\ldots ,n$. However, the reward $r_{t}^{\pi }\left(x\right)\;$ acquired in a sensing period T is computed after n spectrum sensing have been completed. The probability of sensing a vacant channel is $N_{D}$ where $0\leq N_{D}\leq t_{D}$ for $t_{D}\neq 0$. The reward $r_{t}^{\pi }\left(x\right)\;$ calculated after selecting a channel at time t is given by (7):

(7) $$r_{t}^{\pi }\left(x\right)=\;\frac{N_{D}}{t_{D}}$$

**Figure 2:**
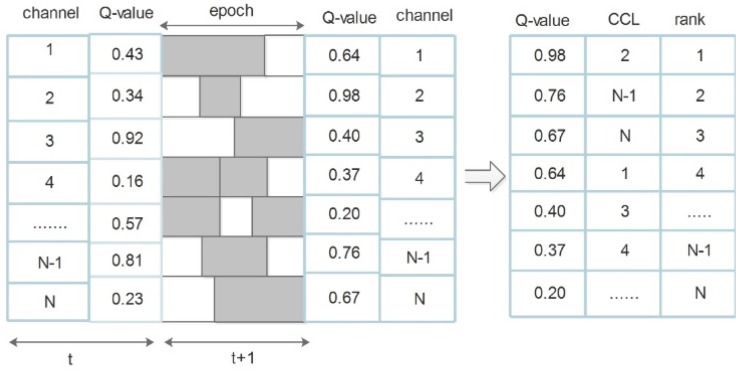
Q-Learning used for channel ranking.

The selection of a channel $C_{i}$ is the action taken at time instant $t\left(a_{t}\right)$ which lasted one sensing period. After learning for a sufficiently long period, Q-values are computed by using the reward $r_{t}\left(a_{t}\right)$ calculated at time t+1. A Q-table is then used to store the computed Q-value. Subsequently, the gateway updates the Q-table through the information it receives in the present state-action tuple $\left(x_{t},\;a_{t}\right)$, including the calculated reward $r_{t}\left(a_{t}\right)$, and the next state $x_{t+1}$ as represented in Figure 2. Then a score is computed for each channels based on the Q-values obtained from the two RL algorithms.

### 4.3. Historical Occupancy Learning

Two criteria are used to calculate the Q-value in the first Q-learning algorithm to reflect the objective of evaluating future channel occupancy as follows [17]: (1) the occupancy rate of the channel in the current sensing period, and (2) the aggregated sum of the occupancy rate in a number of past sensing periods.

If the number of potential channels to be scanned in the sensing period is denoted by ф. Then the Q-value, $Q^{\lambda }\left(c\right)$ of a given channel c of the first Q-learning algorithm in the subsequent sensing period is given by (8) [17,35]:

(8) $$\forall c\in ф⟹Q^{\lambda }\left(c\right)=\left(1-\alpha \right)\overset{l}{\underset{i=1}{\sum }}[w_{t-i}r_{t-i}]\left(c\right)+\alpha r_{t}\left(c\right)$$

where 0≤α≤1 is the unit of the reward $r_{t}$ in the last sensing period which corresponds to the learning rate of the algorithm, l is the number of past sensing periods considered, and w is the unit of each of the l sensing periods.

### 4.4. Channel Conditions Learning

The second Q-learning algorithm takes into account the quality of the channel scanned during the second part of the sensing period to analyze the channels. Therefore, the following criteria which reflect this objective are used to calculate the Q-value [17]: (1) the rate of RSSI in the existing sensing period, and (2) the aggregate sum of the RSSI in a number of past sensing periods l are considered. 

Assuming the number of channels detected to be vacant in the first sensing part is  ф, the RSSI measurements obtained during the second sensing part are used to calculate the Q-value of the second Q learning algorithm. To determine the channel quality, $\;Q^{\psi }\left(c\right)$ for a given channel c in the second Q-learning algorithm is denoted in (9):

(9) $$\forall c\in ф⟹Q^{\psi }\left(c\right)=\left(1-\beta \right)\overset{l}{\underset{i=1}{\sum }}\left[w_{t-1}\eta_{t-1}\right]\left(c\right)+\beta \eta_{t}\left(c\right)$$

where 0≤β≤1 is the unit of the current channel conditions, l is the unit of the channel condition in past sensing periods where channels have been found vacant, and η is a factor of the channel condition corresponding to the reward of the algorithm.

### 4.5. Sorting and Ranking of Channels

Based on the analysis performed in the sensing periods, which is based on the occupancy status and channel condition of the channels analyzed, channels are then sorted and ranked based on a certain score (which is a sum of both Q-values) of each channel for the suitability of the channels for opportunistic transmission. 

If φ is the set number of channel scanned during the sensing period, with their associated Q-values from the two Q learning algorithms. Then, the score $Q^{\kappa }\left(c\right)$ denoting the suitability of a given channel c for opportunistic transmission is given in (10) [17,35]:

(10) $$\forall c\in \varphi ⟹Q^{\kappa }\left(c\right)=\gamma *Q_{t+1}^{\lambda }\left(c\right)+\left(1-\gamma \right)*\;Q_{t+1}^{\psi }\left(c\right)$$

where γ is the historical occupancy weight, and (1−γ) is channel conditions weight respectively.

The channel with the highest score, which is the channel at the top of the list is best channel to deploy for opportunistic communication. Whereas, the channel with the lowest score representing the worst channel for opportunistic transmission is placed at the end of the list (see Figure 2 and Algorithm 1 respectively).

The pseudo-code describing the implementation sequence of the task of selecting and ranking channels for the CCL are described in Algorithm 1.

**Algorithm 1** Selecting and ranking the CCL1
ϕ←SSF(CCL)
2
t=current sensing period
3
for each c ∈ϕ do
4   for i=1 to l do5    Compute Occupancy Profile using Equation (8)6    Compute Channel Conditions using Equation (9)7   end for8
t++
9
end for
10
for each c ∈ϕ do
11   if c is free then12     φ←append(c)13   end If14
end for
15
for each c ∈φ do
16   Compute Score using Equation (10)17
end for
18
R←Rank(φ)


### 4.6. Dimensionality Reduction for Learning Convergence

In the industrial wireless environment, occupancy rate and channel conditions are non-stationary and vary abruptly, resulting in a high-dimensional state space. Therefore, if the full state dimensionality is kept for learning, it can lead to selection of channels with degraded characteristics, and increased computational resources as well as convergence time. To mitigate the curse of dimensionality, only space elements that are relevant to task learning are retained from sensing information to improve the convergence of learning. To achieve this, a method of masking linearly dependent and useless state elements to reduce state dimension is implemented in this work to improve the convergence of Q-learning [30,42]. Basically, the process involves evaluating the linear independence of the state vector, and then eliminating linearly dependent elements. Therefore, as the RL task progresses, assuming a set of state values S are stored in a table $T^{\left(S\right)}$, and X is the data stored at the nth episode of the RL task in the table $T^{\left(S\right)}$. Let X be an m×n matrix with a single data sample in each column. Where n is number of data samples in X, and m is the dimension of each data sample. Thus, as the volume of data increases with each RL episodes, the dimension of $T^{\left(S\right)}$ will continue to increase adding extra computational and memory overhead to the system. Therefore, to improve the convergence of the RL task, and to reduce computational and memory resources, all linearly dependent state elements, which are useless state dimensions to the RL task are eliminated to reduce the curse of dimensionality. To achieve this, the covariance of matrix X is evaluated to validate the data samples first, before checking the linear independence of X. The covariance of matrix X is then calculated by subtracting the mean value of each row from X so as to make each row of matrix X have a zero mean. Hence, if a zero eigenvalue of the covariance matrix of X exist, then, the matrix X has linear dependent dimensions. Thus, all the linearly dependent dimensions of matrix X are eliminated leaving only linearly independent state dimensions which are relevant to the RL task in matrix B. Algorithm 2 explains the process.

**Algorithm 2** Reducing state dimension1Let $T^{\left(Q\right)}:\psi \to Q^{*}\left(\psi \right)$ denotes the Q-table used to store the computed Q-values obtained from Q learning.2Let S-table $T^{\left(S\right)}:s\to V^{*}\left(s\right)$ store the state values for the state S from *Q* learning.3Let X which is a m×n matrix represents a snapshot of the S-table at the nth learning episode, where n is the number of data samples in X, and m is the dimension of S. 4Let matrix B represents only linearly independent state dimension which are relevant to the task learning.5Initialize matrix B to an empty set.6Select and remove x which is a linearly dependent and irrelevant state element of the task learning from X
7If zero eigenvalues of the matrix ${\left[xB\right]}^{T}$ exist, then x is linearly independent with matrix B.8Insert x into matrix B.9Repeat steps (6) to (8) until *X* is empty

A convergence and stopping criteria of the algorithm is inserted in (11) as:

(11) $$\sum_{i=1}^{l}w_{i}=1$$

It is anticipated in (11) that sensing stops or the algorithm converge once the weights $w_{i}$, of the number of sensing l, performed is equal to 1. 

### 4.7. Creating BCL for Each CR Users in the Network

In this paper, we consider a multiple-node IIoT model network. As a result, there is a possibility that one or more CR users performing SH at the same time will select the same channel in R. This may lead to collisions if the same channel is selected by multiple CR users [16,42]. To mitigate such possibility, the gateway synchronizes channel selection for all CR users in the network by creating a BCL for each CR user according to the QoS requirement of each CR user. Since we assume that CR transmission starts in the default channel of a CR user. Suppose there are N CR users transmitting in their default channels and the N CR users are attempting to select channel c∈ φ with the highest score $Q^{\kappa }\left(c\right)$ in R as a target channel. Then, the steps adopted by gateway to mitigate collision are as follows: (1) the gateway starts to estimate a residual idle time $t_{c}$ for each CR user j which by definition [16], is the duration between the time instant the CR user starts utilizing its default channel and the time instant another SH is necessary or desirable by CR user j. (2) The gateway assigns channel c to the CR user j with the highest residual idle time. (3) this procedure is repeated several times since a CR user may need to perform several SHs before completing its transmission. Eventually, a CR user uses the channels that have been assigned to it by the gateway to construct a BCL. Ultimately, each CR user uses channel quality metrics e.g., historical occupancy and channel conditions of its associated BCL to select the best target channel the next time an SH is triggered instead of scanning all the channels in R for target channel.

### 4.8. Conditions for Spectrum Handoff

A sequence of events resulting in SH due to the arrival of a PU on the channel previously utilized by the CR users begins by the CR user sensing the licensed spectrum band [43]. In this paper, the clear channel assessment with energy detection and carrier sensing (CCA-ED-CS) approach is adopted to detect the presence of a PU on the channel. This approach is similar to the one adopted by the IEEE 802.11 (Wi-Fi) standard [44], where a PU signal is detected when a CR node senses an energy above a pre-defined threshold. In (12) and (13), we illustrate how this approach is similar to differentiating between two hypotheses:

(12) $$H_{0}:y_{i}\left(t\right)=n_{i}\left(t\right)$$

(13) $$H_{1}\;:y_{i}\left(t\right)=h_{ij}\left(t\right)x_{j}\left(t\right)+n_{i}\left(t\right)$$

where $y_{i}\left(t\right)$ is the received signal of the CR node i, $x_{j}\left(t\right)$ is the signal transmitted from PU transmitter, $n_{i}\left(t\right)$ is the zero-mean Gaussian noise with variance $\sigma^{2}$ at CR node i, and $h_{ij}\left(t\right)$ is the fading channel coefficient. $H_{0}$ signifies the absence of a PU signal, while $H_{1}$ denotes the presence of the PU signal. Nevertheless, the algorithm proceeds to determine if a transmitting pair of CR nodes needs to perform SH to continue transmission in a new target channel due to the validation of hypothesis $H_{1}$. To achieve this, the CR node senses the energy from the PU transmitter by transmitting a sounding signal between the time intervals $t_{1}$ and $t_{2}$. Then CR user listens between the time intervals $t_{2}$ and $t_{3}$ when its sounding signal is on and off respectively. Afterwards, the energy of the signal from the PU transmitter is measured by the CR user as signifies in (14) and (15) [45]:

(14) $$Y_{1}=\frac{1}{w_{0}}\overset{t_{2}}{\underset{t_{1}}{\int }}{\left|y\left(t\right)\right|}^{2}dt$$

(15) $$Y_{2}=\frac{1}{w_{0}}\overset{t_{3}}{\underset{t_{2}}{\int }}{\left|y\left(t\right)\right|}^{2}dt$$

where $w_{0}$ represents the power spectral density of noise. Based on this spectral awareness, the conditions that CR nodes may perform SH procedure is validated in (16);

(16) $$\{\begin{array}{c} H_{0}\;\;\;\;\;if\;\;\;\;E_{1}>E_{2},\\ H_{1}\;\;\;\;if\;\;\;\;\;E_{1}=E_{2}. \end{array}$$

where *E*_1_ and *E*_2_ are the received powers by CR nodes from the PU transmitter between time intervals $t_{2}$ and $t_{3}$ when its sounding signal is on and off, respectively. Hypothesis $H_{0}$ implies that sensed licensed channel is still available, therefore a transmitting pair of CR nodes do not need to perform SH. whereas, hypothesis $H_{1}$ means the sensed licensed channel is occupied, hence CR nodes need to pause on-going transmission and perform SH immediately to an access new target channel. However, the final decision to switch channel is not based solely on the availability/non-availability of the channel but also on the channel condition in the current channel. Moreover, in industrial environments, channel conditions are extremely time-variant due to environmental influences and heterogeneous networks operating on the same frequency [46]. To determine if the quality of its current channel is no longer acceptable and if it will be requiring that a CR user should initiate SH procedure due to bad channel conditions. In this paper, a CR user must proceed to estimate the impact of channel attenuation on the QoS constraints of CR applications in its current channel. To achieve this objective, a pair of communicating IIoT nodes acquire several values of received signal strength indicator (RSSI) of the industrial-WSN environment from their transceivers to estimate channel quality index $\;C_{A}$ throughout periods with poor packet reception rate as denoted in (17) and (18) respectively:

(17) $$\;C_{A}=1-\;\frac{\sum_{i=0}^{i<n}Q_{i}}{n}$$

(18) $$Q_{I}=\{\begin{array}{cc} 0\; & if\;R_{i}\;<\;\tau \;\\ 1\; & if\;R_{i}\geq \;\tau \; \end{array}$$

where $Q_{i}$ is equal to 0 or 1 depending on the value of τ, where τ, is a threshold adaptively fixed by the algorithm depending on the impact of signal attenuation on the amplitude of the signal in the channel state i, n is the number of signal samples used to calculate $Q_{i}$, and $R_{i}$ is RSSI from the ith signal sample in the channel condition i. 

### 4.9. Decision Making

A SH decision making is a major and fundamental step in every SH procedure/technique. Furthermore, decision making plays a vital role in realizing CR users’ needs, and coordinating network resources as well as applying and making the best use of network resources and network performance [47]. Moreover, every SH scheme is different by the nature of the trigger in the handoff algorithm that enables the activation of SH decision procedure. Therefore, to have a robust SH procedure/technique, it better to combine one or more criteria for handoff decision-making process. In this work, channel condition as well as PU arrival are the two criteria used in decision-making for the SH procedure. Firstly, after it is definite that adaption of link consistently falls below the predefined threshold, the process of SH is triggered, as defined in (19):

(19) $$\{\begin{array}{cc} H_{2} & if\;C_{A}<\;0\;\\ H_{3} & if\;C_{A}\leq \;1 \end{array}$$

hypothesis $H_{2}$ means that prevailing channel conditions meet predefined channel requirements and CR nodes do not perform SH. Otherwise, hypothesis $H_{3}$ implies that prevailing channel condition falls consistently below predefined threshold and CR nodes need to perform SH. The critical decision to determine if a transmitting pair of CR nodes should continue on-going CR transmission in a new channel or remain on current channel is taking by testing for all conditions in (16) as well as (19) as presented in (20). In this paper, based on the occurrence of these conditions, which is totally dynamic and spontaneous, and depends on a lot of factors e.g., behavior of IIoT wireless environments, PU activity pattern, as well as disappearance and appearance of spectrum holes. Our algorithm is designed to produce the best decision which returns the best throughput performance, and with minimal SH latency:

(20) $$\{\begin{array}{cc} H_{2\;} & if\;C_{A}<0,\;\\ H_{3} & if\;C_{A}\leq 1,\\ H_{0} & if\;E_{1}>E_{2},\\ & and,\\ H_{1\;} & if\;E_{1}=E_{2} \end{array}$$

The implementation of the criteria for decision-making and execution of SH are highlighted in the pseudo-code in Algorithm 3.

**Algorithm 3** Conditions and decision for SH 1:Compute the Received Power $E_{1}\;\mathrm{and}\;E_{2}$ of the Channel,2:Obtain the Channel Quality Index, $C_{A}$ using Equation (13)3:
$if\;E_{1}>E_{2}\;and\;\;C_{A}<0\;then$
4:   Continue transmission5:    $else\;if\;E_{1}\leq E_{2}\;and\;\;C_{A}=1\;do$6: Execute handoff to target channel, R, obtained from Algorithm 17: end if

## 5. Simulation Setup

To set up our simulation scenario, we placed the primary network comprising PUs owners of the licensed bands beside the secondary network comprising IIoT devices and other nodes, e.g., Bluetooth devices in a network area of radius 100 m. The IIoT nodes interact in a single-hop star topology in the secondary network with coverage radius of 35 m. A total of 10 IIoT devices with three channels was simulated in the secondary network to generate congestion. We used the statistical channel models in (5) considering mainly lognormal shadowing effects and zero-mean Gaussian distributed channels to capture attenuations. We simulated an obstructed industrial environment with a propagation-loss exponents ($2\leq n_{p}\leq 3$), and path loss of 20 dBm. The packet rate of the IIoT device’s transmission follows a Poisson distribution. The interaction of the IIoT devices in the secondary cell is autonomous of the primary network. However, due to the congestion created in the secondary network, the IIoT devices are assumed to opportunistically deploy the licensed band for CR packet transmission. We assume that the IIoT devices are aware of the statistical history of PU’s communication pattern through the gateways, therefore, we simulated a deterministic traffic model in the primary network with a coverage radius of 50 m. The distance between the transmitter of the IIoT devices and the receiver of the PU have been employed for bounding the interference threshold as described in (3) and (4). Therefore, the SINR (1–15 dB) in the primary network are simulated as a function of the random distance of the IIoT devices and/or PUs. The transmit power of the IIoT devices are set at −63 dBm. In this work, we investigate the performance of the proposed SH scheme by extensive MATLAB simulations. In Table 2, the simulation setup is presented.

## 6. Simulation Results

In this section, we compare the latency and throughput performances of the SH+RL scheme with five SH schemes: a reactive SH with sequential spectrum sensing (RSHSS) [16], an SH management scheme with a QoE-driven channel allocation strategy [30], the conventional reactive SH scheme with random selection strategy (RRSS) (in this scheme, target channels are selected randomly from the outcome of an instantaneous spectrum sensing after an SH triggering event has occurred), the conventional proactive SH scheme with random selection strategy (PRSS) (in this scheme, target channel are selected randomly from spectrum sensing outcome before an SH triggering event occurs) respectively. In this paper, latency is defined as the period of time between the instant an SH request is initiated and the instant a CR node is connected to a new channel. Likewise, throughput represents data rate of CR node during CR transmission. During these periods, several interruptions may occur due to low SINR and/or the arrival of PU on the channels. To evaluate the performance of our proposed algorithm (the SH+RL scheme) in diverse IIoT scenarios, each performance metrics has been evaluated as a function of the following parameters i.e., bandwidth size, SINR level, as well as spectrum occupancy as is typical for different IIoT scenarios. Similarly, for better convergence of our results, each point on the graphs has been averaged over 1000 Monte Carlo simulations. Unlike single-point-estimate performance analysis, Monte Carlo simulation is usually used to increase results accuracy, as well as to improve sensitivity and convergence of performance metrics.

**Figure 3:**
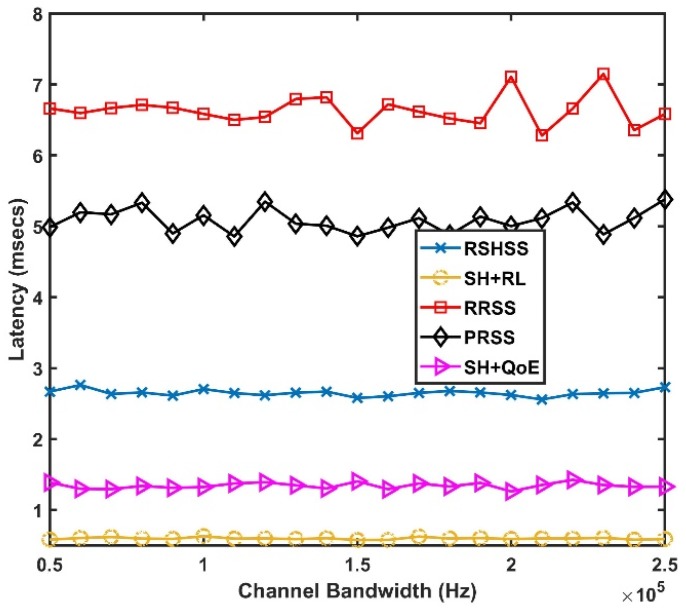
Latency as a function of channel bandwidth.

Figure 3 shows the latency of the five SH schemes under different channel bandwidth. As shown in Figure 3, the SH+RL scheme outstrips other SH schemes in terms of lower latency. This significant reduction in latency is attributed to the fact that in the SH+RL scheme, a new target channel is always available in the BCL assigned to each CR user, and the time used to scan the associated BCL of each CR user is always shorter than the time used to scan the whole CCL/the entire frequency band in the SH+QoE, and the RSHSS schemes respectively. Therefore, the time spent in selecting a target channel and switching to the new channel is always shorter for the SH+RL scheme than for the SH+QoE, as well as the RSHSS schemes, which results in lower latency. The random selection strategy in the reactive and proactive modes (i.e., RRSS and PRSS schemes) lead to approximately the same performance. This can be attributed to the fact that channel selection for an SH is done randomly in both the RRSS and PRSS schemes. This leads to a high possibility of selecting a channel occupied by the PU all the time. The time spent in rescanning the channel for an unoccupied channel leads to the observed higher latency in both the RRSS and PRSS schemes. However, in relative terms, the PRSS scheme has a slightly better latency performance than the RRSS scheme because spectrum sensing and channel switching is done before the PU appears. The observed lower latency in Figure 3 for the SH+RL, the SH+QoE, and the RSHSS schemes in comparison to the RRSS and the PRSS schemes highlights the importance of keeping a CCL, in minimizing interference to the PU, in reducing number of times the channels is rescanned for unoccupied channel, and ultimately in minimizing latency.

**Figure 4:**
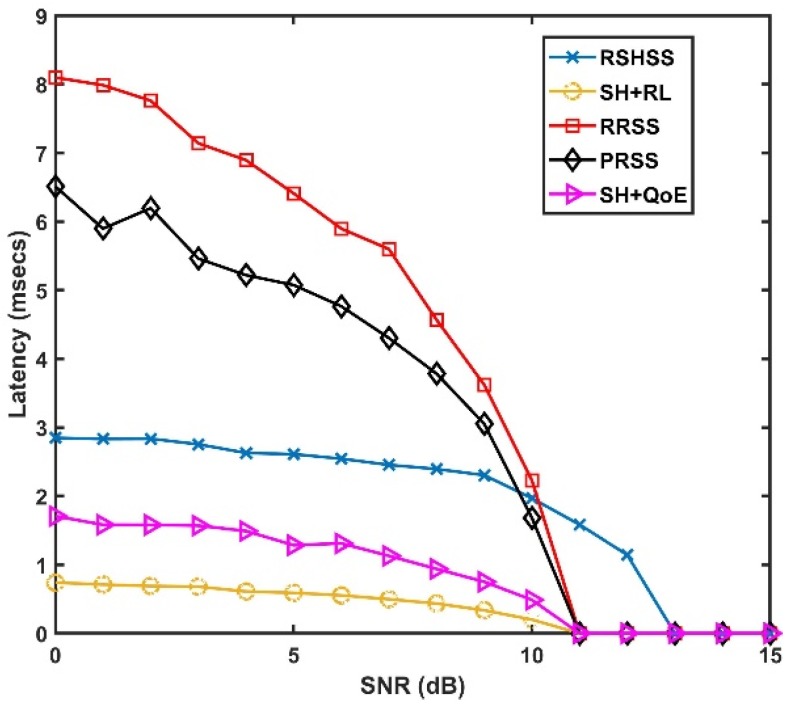
Latency as a function of SNR.

Figure 4 presents the latency of the five SH schemes at different SNR values. Similar to the result presented in Figure 3, it can be observed that the SH+RL scheme has the lowest latency while the RRSS and the PRSS schemes have the highest latency with respect to varying SNR values. The closest to the SH+RL scheme in terms of latency performance is the SH+QoE scheme. While in the SH+RL scheme, the RL algorithm gives more weight to past sensing periods than in the current sensing periods, in the SH+QoE scheme channel selection is based solely on the current occupancy profile of the channels. This approach does not reflect the actual historical occupancy profile of the channel. Therefore, the sorting and ordering of the CCL is outdated at the time of SH, resulting in high latency than in the SH+RL scheme. Conversely, the approach used in the SH+RL scheme, leads to a higher probability of accurate profiling of the channels in terms of occupancy, as well as a higher probability that the channels will always be available at the time of SH, which results in lower latency. Although, the RSHSS scheme performs better than both the RRSS and PRSS schemes, yet, the SH+RL scheme still outperforms the RSHSS. This is because, in the RSHSS scheme, an SH procedure is only initiated in response to an SH triggering event. As a result, the target channel is selected from the outcome of an instantaneous wideband sequential sensing of the frequency spectrum. This leads to high latency due to spectrum sensing and reconfiguration delays. 

**Figure 5:**
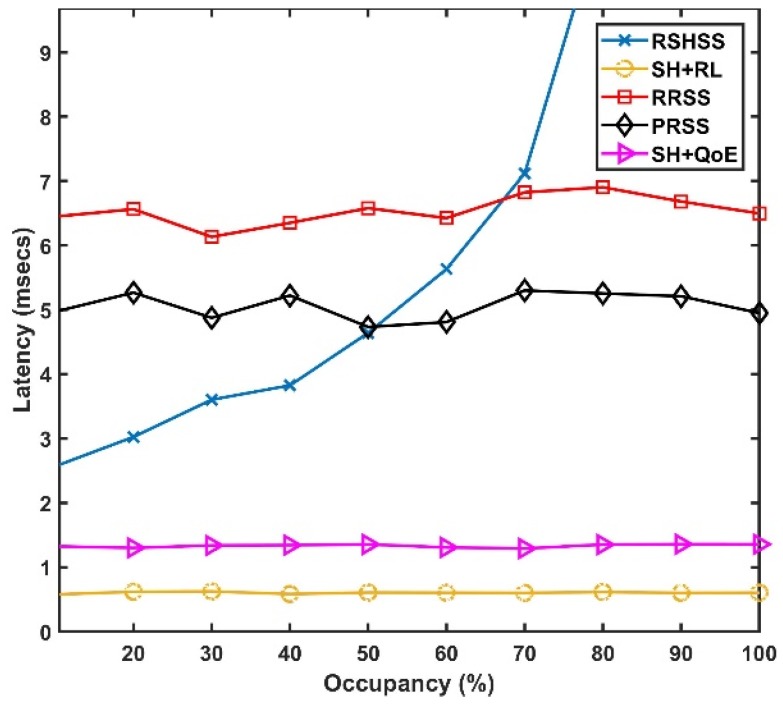
Latency as a function of Occupancy.

In Figure 5, we plot latency as a function of the percentage of channel occupancy for the five SH schemes. Clearly, from Figure 5, the RRSS and PRSS schemes have the poorest latency performance at lower channel occupancy. However, the RSHSS scheme has the highest latency as channel occupancy increases. This is because, an increase in channel occupancy increases the probability that a PU signal will be detected in most of the channels. Therefore, it becomes increasingly difficult to find a new channel by sequentially scanning the frequency spectrum resulting in the exponential increase in latency in RSHSS scheme. Still, the SH+RL scheme has lower latency than the SH+QoE schemes with increasing occupancy. This is due to the fact that in the SH+RL scheme, the RL algorithm is always able to create a CCL and BCL by historical occupancy learning. Therefore, a new target channel is always available during the time instant of SH in the SH+RL scheme resulting in the lowest latency. Whereas, in the SH+QoE scheme, it becomes increasingly difficult to create a CCL as channel occupancy is increasing. This is because sorting and ordering of channels are based solely on the current occupancy profile of the channels. 

**Figure 6:**
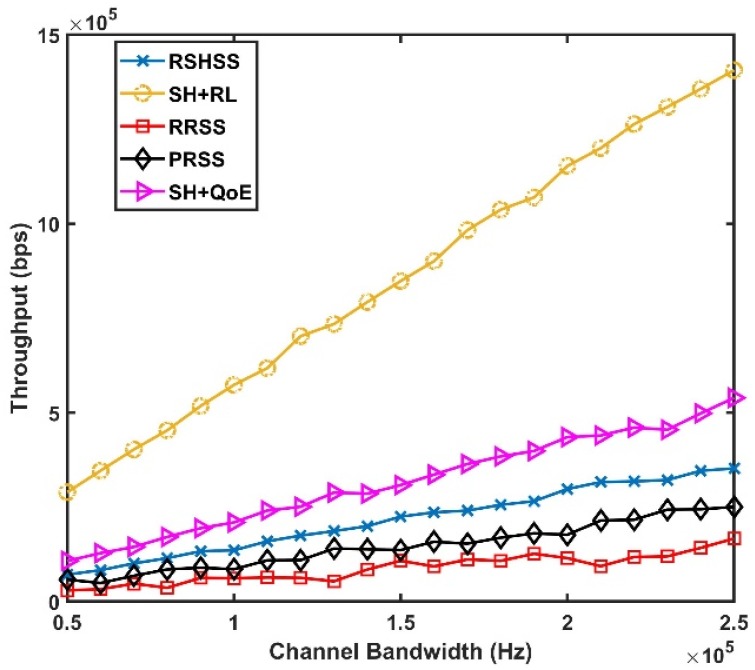
Throughput Performance as a function of Channel bandwidth.

The throughput performance in Figure 6 shows a major improvement by the SH+RL scheme. This is because, unlike in the RRSS, and PRSS schemes respectively, in the SH+RL scheme, channels are not simply selected randomly in the CCL, as well as the BCL, based on the availability of the channels alone. But, the channels are selected based on the channel quality score, in addition to being vacant/unoccupied/available leading to improved throughput performance when CR users utilize such channels for CR transmission as illustrated in Figure 6. Similarly, as bandwidth increases, as shown in Figure 6, there is a clear upswing improvement in terms of throughput performance by the SH+RL scheme in comparison to the SH+QoE, and the RSHSS schemes respectively. This is because, through the Channel Conditions Learning algorithm, the best available channels are allocated to CR users during handoffs in the SH+RL scheme. Therefore, having the best channels for transmission has resulted in the comparative differential improvement in terms of throughput performance for the SH+RL scheme as observed in Figure 6.

**Figure 7:**
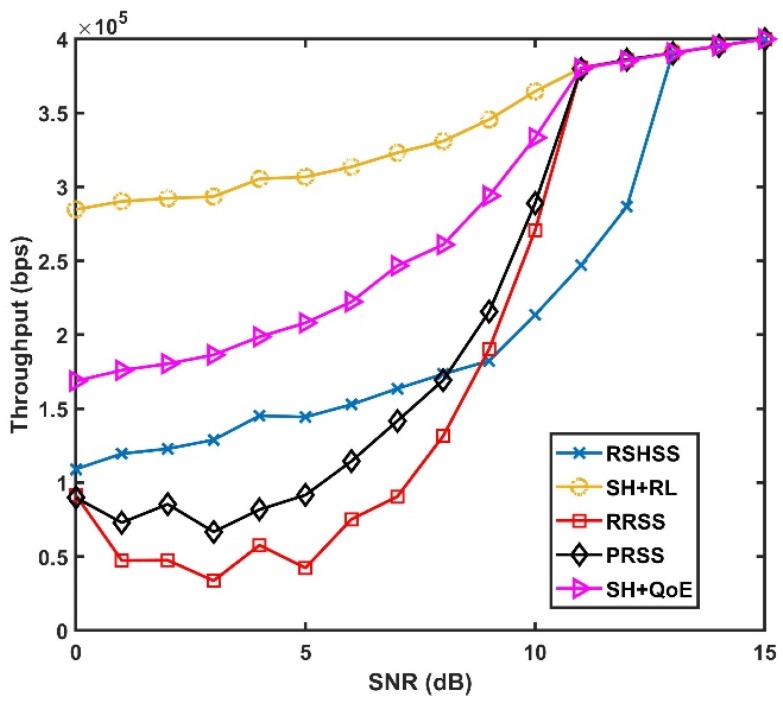
Throughput Performance as a function of SNR.

Figure 7 compares the throughput performance of the five SH schemes at different SNR values. As shown in Figure 7, the SH+RL scheme outperforms other SH schemes. This is because, owing to the RL algorithm executed in the SH+RL scheme, a target channel with sufficiently good channel quality is always available at the time of SH leading to a comparatively better throughput performance. However, it can be observed in Figure 7, that as SNR increases, the throughput performance of the SH+RL scheme converged with the throughput performances of the SH+QoE, the RRSS and the PRSS schemes at 11 dB and the RSHSS scheme at 13 dB, respectively. Therefore, it does appear that the SH+RL scheme may not outperform other SH schemes in high SNR values. In reality, however, extremely good channel conditions are unrealistic in the industrial wireless environment due to the time and link varying nature IIoT environment, so the result in Figure 7, further validates the ability of the SH+RL scheme to maintain better throughput performance in the extremely harsh industrial wireless environment illustrated in the lower SNR values.

**Figure 8:**
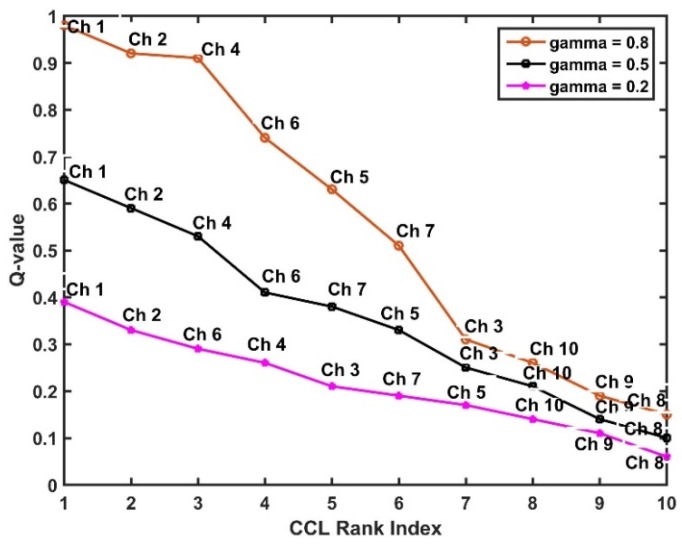
Effect of gamma on channel list ranking.

The results in Figure 8 show how varying weights of the channel occupancy learning and channel condition learning, respectively, can affect the ranking of the CCL. We set the values of  λ=0.8, 0.5, and 0.2 to simulate scenarios where either of the weights is higher than the other, and the third scenario, where both weights have the same value, respectively. It can be observed in Figure 8, that channels 1 and 2 remain the two best channels in all three setups. Whereas, the channels at the bottom (channels 10, 9, and 8) of the CCL, even though retained their positions in all different setups remain the worst channels. However, the channels in the middle (channels 3, 5, and 7) are the most affected, with channel 5 swapping position from 5th to 6th and finally to 7th position in the three different setups. This is because, the RL is a reward-based task, and as a result, if a small reward is obtained during the task learning a small Q-value is calculated. Nonetheless, the general trend observed in Figure 8 is that the Q-value of all the channels (channels 1 to 10) reduces as the value of λ decreases in the different setups, this is due to the fact that channel condition fluctuates more than channel occupancy as the weights decreases, thus, affecting the Q-values. Therefore, we can conclude that weights should be defined in relation to the behaviour of the radio environment. For example, in noisy environment, e.g., IIoT, small values should be given to λ, so as to give more significance to channel condition learning. Whereas, in environments with good signal-to-noise ratio, λ can be given higher values, in order to give more prominence to the channel occupancy learning task.

## 7. Conclusions

In this paper, we have developed an SH scheme for the IIoT that specifically takes into consideration the design of a CCS strategy for implementing the handoff scheme. Moreover, because our objective is to design a CSS strategy that jointly maximizes channel availability and channel conditions for an effective SH procedure, with the intent to reduce SH latency and to improve throughput performances for IIoT systems and industrial application scenarios. Therefore, we specially implemented an RL algorithm in the operation of the CCS strategy. This is to optimize the sorting and ordering of channels in the CCL, and also to take into account channel availability as well as channel conditions as key performance metrics, specifically in IIoT. Moreover, it is also to reduce the time spent in the instantaneous wideband scanning of the entire licensed spectrum band for the availability of prospective new target channels. As well as to reduce the likelihood of selecting a channel with a bad channel quality if target channel is selected randomly during an SH procedure. We investigated the performance of the proposed SH scheme by extensive MATLAB simulations of the industrial wireless environment. Remarkably, the results showed a significant reduction in terms of latency performance while noticeably improving throughput performance by the SH+RL scheme in comparison to other SH schemes.

## Figures and Tables

**Table 1:** Real-time and QoS requirements of the industrial applications.

Industrial System	Categories	No. of Nodes	Real-Time Requirements	QoS Requirements	Latency (ms)	Reliability (PLR)	Jitters (ms)
**Monitoring systems**	Information and Alerting systems	100–1000	No real-time	Reliability, energy efficiency, load balancing, and availability	≥100	10^−3^–10^−4^	−
**Safety systems**	Alarm systems	100–300	Soft real-time	Availability, also timeliness	10–100	10^−3^–10^−4^	≤1
**Control systems**	Control and Factory automation	2–50	Hard real-time	Timeliness, reliability, and energy efficiency	0.25–10	10^−9^	≤0.02

**Table 2:** Simulation setup.

Parameter	Value
Unlicensed band	
Frequency	2.4 GHz ISM band
Transceiver	CC2420
Number of active nodes	10 IIoT devices
Bandwidth	50 KHz
Number of channel	3
Packet rate	Poisson distribution
SINR	5 dB
Licensed band	
Frequency	470–890 MHz
Number of active nodes	5 PUs,
Number of channel	10
Packet rate	Poisson distribution
SINR	1–15 dB
Network and channel model	
Network radius	100 m
PU coverage radius	50 m
IIoT devices coverage radius	35 m
Propagation-loss exponent	Random $2\leq n_{p}\leq 3$ (obstructed industrial environment)
Transmitted power	−63 dBm
Path loss	20 dBm
